# Impact of quality circles for improvement of asthma care: results of a randomized controlled trial

**DOI:** 10.1111/j.1365-2753.2007.00827.x

**Published:** 2008-04

**Authors:** Antonius Schneider, Michel Wensing, Kathrin Biessecker, Renate Quinzler, Petra Kaufmann-Kolle, Joachim Szecsenyi

**Affiliations:** 1 Senior Researcher, Department of General Practice and Health Services Research, University Hospital of HeidelbergHeidelberg, Germany; 2 Senior Researcher, Department of General Practice and Health Services Research, University Hospital of HeidelbergHeidelberg, Germany; 3 Research Assistant, Department of General Practice and Health Services Research, University Hospital of HeidelbergHeidelberg, Germany; 4 Research Assistant, Department of Clinical Pharmacology and Pharmacoepidemiology, Medical Clinic and Policlinic, University Hospital of HeidelbergHeidelberg, Germany; 5 Pharmacist, AQUA-Institute for Applied Quality Improvement and Research in Health CareGöttingen, Germany; 6 Professor, Head of the Department and AQUA-Institute, Department of General Practice and Health Services Research, University Hospital of HeidelbergHeidelberg, Germany

**Keywords:** asthma, emergency visit, general practice, hospitalization, quality circle, self-management

## Abstract

**Rationale and aims:**

Quality circles (QCs) are well established as a means of aiding doctors. New quality improvement strategies include benchmarking activities. The aim of this paper was to evaluate the efficacy of QCs for asthma care working either with general feedback or with an open benchmark.

**Methods:**

Twelve QCs, involving 96 general practitioners, were organized in a randomized controlled trial. Six worked with traditional anonymous feedback and six with an open benchmark; both had guided discussion from a trained moderator. Forty-three primary care practices agreed to give out questionnaires to patients to evaluate the efficacy of QCs.

**Results:**

A total of 256 patients participated in the survey, of whom 185 (72.3%) responded to the follow-up 1 year later. Use of inhaled steroids at baseline was high (69%) and self-management low (asthma education 27%, individual emergency plan 8%, and peak flow meter at home 21%). Guideline adherence in drug treatment increased (*P* = 0.19), and asthma steps improved (*P* = 0.02). Delivery of individual emergency plans increased (*P* = 0.008), and unscheduled emergency visits decreased (*P* = 0.064). There was no change in asthma education and peak flow meter usage. High medication guideline adherence was associated with reduced emergency visits (OR 0.24; 95% CI 0.07–0.89). Use of theophylline was associated with hospitalization (OR 7.1; 95% CI 1.5–34.3) and emergency visits (OR 4.9; 95% CI 1.6–14.7). There was no difference between traditional and benchmarking QCs.

**Conclusions:**

Quality circles working with individualized feedback are effective at improving asthma care. The trial may have been underpowered to detect specific benchmarking effects. Further research is necessary to evaluate strategies for improving the self-management of asthma patients.

## Introduction

Asthma has a high prevalence as a chronic disease, affecting nearly 5% of the population in industrialized nations [1]. It is a comparatively cost-intensive illness because of chronic medication and frequent hospitalization accompanied by periods of disability. In Germany, total costs have been estimated at €2.74 billion for 1999 [2]. The hallmarks of effective therapy to stabilize the course of disease are optimal medication and self-management. Medication is optimal when patients use inhaled steroids [3], but this cannot be taken for granted, as not only patients [4] but also doctors might have some aversion to steroids [5]. The optimal self-management of patients comprises asthma education and keeping an asthma diary, and the regular measurement of peak expiratory flow. These measures have been demonstrated to be effective at reducing morbidity and mortality [6]. However, many patients seem to feel uncomfortable with regular monitoring of the disease [4,7] and thus need to be repeatedly motivated for self-management. Therefore, general practitioners (GPs) have a key role in continuous support, as they have to provide comprehensive medical care. This involves manifold duties and responsibilities, such as organizing education, providing asthma diaries, disease monitoring and medication control.

Quality circles (QCs) are well-established instruments for aiding a doctor's daily work by regular conjointly reflection on common practice with other colleagues [8]. However, one large trial evaluating the group education of GPs identified only small improvements in asthma and chronic obstructive pulmonary disease (COPD) care [9]. Another trial found peer-review groups combined with individual feedback to be only slightly effective for asthma care [10]. In QCs using individual feedback, prescribing data are discussed in the group under the guidance of a moderator without exchanging individual practice results [11]. New quality improvement strategies include feedback with benchmark activities, where ‘best practice’ is openly labelled and discussed [12,13]. However, it is unclear whether the presentation of ‘achievable benchmarks of care’ is effective for QCs. The aim of the trial was to evaluate (a) the effectiveness of QCs using the individualized feedback of prescribing data, and (b) the effectiveness of benchmarking on asthma care, in particular on asthma severity as the most patient-relevant outcome parameter. A secondary aim was to evaluate the impact of medication guideline adherence on hospitalization and unscheduled emergency visits in primary care.

## Methods

### Study design

The study was designed as a two-armed randomized controlled trial. One arm comprised a traditional QC without benchmark. The second arm comprised a QC working with an open benchmark and discussion of the results. After giving consent, the GPs were randomized to the ‘traditional’ arm vs. the study arm working with benchmarking. These GPs were grouped into a total of 12 QCs. Six QCs worked with an open benchmark, and the other six worked with the traditional discussion style. The study was approved by the Medical Ethics Committee of the Medical Faculty of the University of Heidelberg on 12 November 2004 (application number 371/2004).

### Traditional QCs

An individual feedback report about prescribing data from one's own practice is normally posted to each doctor a few days before they meet in QCs, so that the report can be read before the meeting. In the QCs, the doctors usually interpret their own data without exchanging their individual practice results with one another. The GPs also receive a feedback report with additional information about the group performance of the other doctors. This allows them to compare their own results with the variability of performance (median, quartiles, minimum and maximum) of the other doctors. The GPs discuss problems of care under the guidance of a moderator, and the respective quality indicators serve as a basis for discussion. In the present study, the GPs in traditional QCs also received the baseline questionnaire results describing the number of patients with an individual emergency plan, an asthma diary and peak flow meter at home. Using this information, the doctors discussed the feasibility of a new set of asthma guidelines, the management of asthma education or prescribing management. The discussion was facilitated by comprehensive information on evidence-based asthma therapy, including examples of individual emergency plans and asthma diaries, and other indices of patient management.

### QCs with benchmarking

The basic structure is identical to that of traditional QCs: individual feedback reports were given to the participating GPs in these QCs, but with a comparison with a performance ‘benchmark’. These GPs received the name of and information about the GP who performed best in their QCs and information about the performance of the ‘best 10%’ of GPs in the benchmark arm. Under the guidance of the moderator, the GPs discussed with the identified GP how the best practice was achieved. In addition, practice details of the ‘overall best practice’ of the benchmarking arm were given to enable a comparison with the best benchmark. This multifaceted benchmark intervention was intended to allow learning from the best performer. The moderators of both arms were trained before the QC meetings to lead the group discussion.

### Setting and patients

At the beginning of this study, 97 GPs in 87 general practices collaborating in this project were asked to participate in the survey. As the workload in these practices was particularly high because of the implementation of a new fee-for-service structure during the study period, only 42 practices agreed on handing out the questionnaires (27 in the benchmarking group and 15 in the traditional group). The GPs were instructed in a leaflet to select patients with asthma as accurately as possible. The most relevant criteria to distinguish between asthma and COPD were explained in this leaflet. For example, the GPs were instructed to preferably select patients with varying symptoms, attacks of dyspnoea and wheezing, or with a known allergy. They were to avoid including heavy smokers, who were likely to have COPD.

The GPs were asked to hand out a questionnaire consecutively to every asthma patient coming for consultation between May and July 2005 (T1). The patients were asked to fill in the questionnaire and to send it to the study centre, receiving assurances of the anonymous handling of their data. Three lots of €250 were raffled as an incentive for participation. One year later (T2), the patients received the same questionnaire directly from the study centre. Again, three lots of €250 were raffled as an incentive. Three reminders were sent out after 4, 8 and 12 weeks.

### Measures

Routine prescribing data as primary outcome measures are yet to be made available by the compulsory health insurance organization Allgemeine Ortskrankenkasse (AOK). However, these data will be obtained after careful control and anonymizing in February 2007 at the earliest. The secondary outcome measures were evaluated using the questionnaire.

The patients were asked about their daily and nocturnal symptoms related to asthma, according to the international levels of asthma severity steps 1 to 4 [14]. Current medication, including dosage, was documented in a structured register. Furthermore, the patients were questioned on various aspects of self-management. Specifically, we wanted to know whether a patient (a) had already participated in an education programme on asthma; (b) wanted to receive education; (c) had a peak flow meter at home; (d) used a peak flow meter regularly to monitor the disease; (e) had a personal emergency plan; (f) had been admitted to hospital within the last 12 months because of asthma; and (g) had received unscheduled home visits from a GP or ambulatory care due to asthma within the last 12 months.

### Analysis

The questionnaires were scanned and the data were imported automatically into spss 14.0. by Eyes & Hands® Forms, Version 5 (ReadSoft AB, Sollentuna, Sweden). The plausibility of the data was checked manually, and baseline data were presented descriptively. Differences between men and women, and responders and non-responders were calculated with *t*-tests or χ^2^-tests where appropriate.

To assess adherence to guidelines, the medication was checked manually for each patient. Full adherence to guidelines implied that the prescribed medication was consistent with the guidelines [14] and that the patient was in asthma step 1 or 2 for day and night. Guideline adherence but under-dosing of medication implied that the patient received appropriate medication, such as inhaled steroids, but rated him/herself as being in asthma step 3 or 4 for day or night so that the dose could be increased. Inappropriate prescribing of medication implied that the patient had not been treated according to guidelines, for example if only sympathomimetics were given without steroids in step 2, 3 or 4.

Before–after differences of guideline adherence and asthma steps were calculated using the McNemar test, a non-parametric test for dependent samples. Predictive values related to hospitalization and unscheduled emergency visits were estimated using univariate logistic regression.

## Results

### Baseline characteristics and follow-up

A total of 314 patients, of whom 159 (62.5%) were female, received the questionnaire; the average age was 56.8 years. Two hundred and fifty-six (81.5%) patients sent back the questionnaire. Of the responders, 158 (61.7%) were female, and the average age was 56.3 years. There was no significant difference between responders and non-responders with regard to sex and age at baseline (data not shown). Nearly half of the patients reported symptoms corresponding to asthma step 3 or 4 (Table 1). The use of steroids was comparatively high, with 68.8% receiving inhaled and 8.2% oral steroids. Only 27% had previously participated in an asthma programme. The extent of self-management was low, as only 21.1% had a peak flow meter at home, only 4.3% used an asthma diary, and 8.2% had an individual emergency plan.

**Table 1:** Baseline characteristics at baseline (*n* = 256)

	Number of patients (%)
Asthma step at day	
1 Symptoms less than once a week	59 (23.3)
2 Symptoms more than once a week but less than once a day	63 (24.6)
3 Symptoms daily, but not continuously	92 (35.9)
4 Symptoms continuously with limitation to physical activities	19 (7.4)
Medication	
Inhaled sympathomimetics	200 (78.1)
Inhaled steroids	176 (68.8)
Oral steroids	21 (8.2)
Theophylline	85 (33.2)
Montelukast	17 (6.6)
Self-management	
Asthma education	69 (27.0)
Peak flow meter at home	54 (21.1)
Use of asthma diary	11 (4.3)
Individual emergency plan	21 (8.2)
Smoking	65 (25.4)

One hundred and eighty-five patients (72.3%) responded to the follow-up 1 year later. Seventy-one were unable to be included at follow-up. Of these patients, 11 had an unknown address and one patient had died. There was no significant difference in sex, age and self-management between responders and non-responders at follow-up.

### Effects on asthma severity and medication guideline adherence

Clinical improvement was operationalized as change in asthma severity (Table 2). The number of patients with asthma step 3 decreased (*P* = 0.046) and patients with asthma step 2 increased (*P* = 0.055). The non-responders at follow-up seemed to be slightly healthier, as they had more favourable asthma steps; indeed the difference was not significant (*P* = 0.124; χ^2^-test; data not shown). As an overall effect, the change from the unfavourable group (asthma steps 3 and 4) into the beneficial group (asthma steps 1 and 2) was significant (*P* = 0.018). There was no group difference between benchmarking and traditional QCs in all of these categories.

**Table 2:** Changing in asthma steps (before–after comparison)

	Traditional (T1 and T2 complete)		Benchmark (T1 and T2 complete)			
				
Asthma step	T1	T2	T1	T2	*P* (McNemar)	Non-responder at T2
Step 1 (best)	15 (24.2%)	15 (23.8%)	31 (27.7%)	13 (22.0%)	0.097	13 (22.0%)
Step 2	16 (25.8%)	20 (31.7%)	24 (21.4%)	23 (39.0%)	**0.055**	23 (39.0%)
Step 3	27 (43.5%)	21 (33.3%)	46 (41.1%)	19 (32.2%)	**0.046**	19 (32.2%)
Step 4 (worst)	4 (6.5%)	7 (11.1%)	11 (9.8%)	4 (6.8%)	0.541	4 (6.8%)
Total	62	63	112	59		59

T1 = baseline; T2 = follow-up 12 months later. Bold values indicate significant results.

The improvement in asthma severity is slightly reflected in the improvement of asthma therapy. There was a trend towards an increase of full adherence to guidelines and decrease of under-dosing (Table 3). However, these effects were not significant.

**Table 3:** Guideline adherence with therapy (before–after comparison)

	Traditional quality circle (*n* = 62)		Benchmark quality circle (*n* = 113)		
			
Guideline adherence	T1	T2	T1	T2	*P* (McNemar)
Full adherence to guidelines	21 (33.9%)	26 (41.9%)	45 (40.2%)	49 (43.4%)	0.188
Full adherence to guideline, end-of-dose	6 (9.7%)	4 (6.5%)	8 (7.1%)	8 (7.1%)	0.791
Guideline adherence, but dose too low	23 (37.1%)	18 (29.0%)	38 (33.9%)	32 (28.3%)	0.268
No guideline adherence, patient in step 1 or 2	7 (11.3%)	6 (9.7%)	7 (6.3%)	9 (8.0%)	1.000
No guideline adherence, patient in step 3 or 4	5 (8.1%)	8 (12.9%)	14 (12.5%)	15 (13.3%)	1.000

T1 = baseline; T2 = follow-up 12 months later.

### Self-management, hospital stay and unscheduled visits

There was no change in asthma education, peak flow meter at home and use of asthma diary (Table 4). The number of patients with an individual emergency plan increased (*P* = 0.008), although this number remains low. There was a decrease in the number of patients with unscheduled emergency visits (*P* = 0.064) and a decrease in the number of visits (*P* = 0.096). Also, in these categories, there was no group difference between benchmarking and traditional QCs.

**Table 4:** Changes in self-management, hospital stays and unscheduled visits (before–after comparison)

	Traditional quality circle (*n* = 62)		Benchmark quality circle (*n* = 113)		
Management aspects	T1	T2	T1	T2	*P* (McNemar)
Asthma education	20 (30.3%)	20 (30.3%)	37 (31.1%)	39 (32.8%)	0.584
Peak flow at home	16 (24.2%)	20 (30.3%)	27 (22.7%)	27 (22.7%)	0.424
Asthma diary	5 (7.6%)	6 (9.1%)	3 (2.5%)	6 (5.0%)	0.289
Emergency plan	4 (6.1%)	7 (10.6%)	8 (6.7%)	17 (14.3%)	**0.008**
Hospital stay	5 (7.6%)	3 (4.5%)	9 (7.6%)	7 (5.9%)	0.727
Emergency visits	13 (19.7%)	4 (6.1%)	21 (17.6%)	13 (10.9%)	**0.064**
Number of unscheduled visits	14	20	51	20	**0.096**

T1 = baseline; T2 = follow-up 12 months later. Bold values indicate significant results.

### Predictors of emergency visits and hospitalization

The risk for hospitalization and unscheduled emergency visits over 1 year increased significantly with asthma severity (Table 5). The number of patients with unscheduled emergency visits decreased significantly with guideline adherence (‘Guideline adherence’ comprised patients with ‘full adherence to guidelines’ and ‘guideline adherence, end-of-dose’.) The use of theophylline predicted hospitalization and unscheduled visits. An additional logistic regression, including interaction analysis, showed a marginal but non-significant interaction between asthma severity and the use of theophylline with regard to emergency visits (OR for interaction 0.18; 95% CI 0.03–1.30; *P* = 0.09).

**Table 5:** Predictors of unscheduled emergency visits and hospitalization within following 12 months (logistic regression)

	Hospital stay (Yes/No) (T2)			Emergency visit (Yes/No) (T2)		
		
Predictor (T1)	OR	95% CI	P	OR	95% CI	P
Asthma step	2.56	1.07–6.16	**0.035**	3.51	1.63–0.752	**0.001**
Guideline adherence (yes/no)	0.562	0.14–2.33	0.426	0.24	0.07–0.89	**0.033**
Using sympathomimetics	2.03	0.25–16.64	0.509	3.07	0.47–29.10	0.213
Using inhaled steroids	1.48	0.30–7.24	0.629	1.19	0.37–3.87	0.769
Using oral steroids	2.16	0.75–6.20	0.152	0.98	0.27–3.58	0.975
Using theophylline	7.1	1.5–34.3	**0.016**	4.9	1.6–14.7	**0.005**

T1 = baseline; T2 = follow-up 12 months later. Bold values indicate significant results.

## Discussion

The results of our trial indicate that QCs can be effective at improving outcomes for patients with asthma. While asthma steps improved significantly, which is slightly reflected in the improvement of drug treatment, few changes in clinical management were identified. Unscheduled emergency visits decreased, which might be associated with the effect on asthma severity. However, it was not possible with this randomized trial to demonstrate the superiority of using an open benchmark in QCs.

Nearly 70% of patients received inhaled steroids and 8.2% had oral steroids. This is a high level of inhaled steroid usage compared with a huge European survey in which only 23% used inhaled steroids regularly [15]. The quality of medical treatment improved after the QC sessions as full adherence to guidelines increased and under-dosing decreased after 12 months. This effect was not significant, but it is possibly reflected by the significant clinical improvement in patients. In contrast to a postulated under-use of steroids [15,16], our results suggest a comparatively satisfying level of steroid use. It might be overambitious or even inadequate to achieve the goals set by the guidelines, which are reached by consensus [17], in every patient. However, medication with guideline adherence is of importance, as our results illustrate. Patients with optimal medication at baseline had significantly lower emergency visits within the following year.

The association between use of theophylline, hospital stay and emergency visits remains to be discussed. On the one hand, this could indicate low guideline adherence in terms of prescribing, accompanied by under-use of inhaled steroids, which are vital to efficient asthma therapy [1] as they reduce morbidity and mortality [3]. On the other hand, it cannot be excluded that theophylline itself is responsible for emergency cases by way of insufficient suppression of eosinophilic inflammation or by causing serious adverse effects due to its high potential for side effects [18]. Further evaluation would be necessary with larger sample sizes, as the amount of hospitalization is low, thus leading to large confidence intervals and difficulties in adjusting for asthma severity.

Another critical point is the low number of patients receiving education and support for self-management. The relevance of these measures for reducing disability and hospitalization has been demonstrated in several studies [6]. Meng *et al*. also found a low usage of peak flow meter and therefore suggested the dissemination of guidelines to patients themselves [19]. However, two-thirds of the patients in our trial did not want to participate in asthma education, irrespective of asthma severity [7]. Therefore, it remains a challenge for GPs to motivate these patients to participate in asthma education in order to prevent a severe deterioration of the disease. With the exception of the delivery of individual emergency plans, there was no remarkable improvement in these aspects.

However, all these measures together seemed to improve the health outcomes in terms of asthma severity and unscheduled emergency cases. This contrasts with the results of Smeele *et al*., who found group education not effective [9]. One reason for this difference could be that Smeele *et al.* included patients with asthma and COPD, following the ‘Dutch hypothesis’, which presumes a common aetiological factor in both diseases [20]. Newer developments aim to distinguish between these patients, and COPD patients are more difficult to treat because of nicotine dependency and lack of medication to influence the decline of lung function [21]. Therefore, it seems likely that improvement of care is more difficult to measure with changes in COPD patients. This could be the same difficulty in the trial carried out by Jans *et al.*, who tried to detect improvement of peak flow variability in asthma and COPD patients by improving care with a multifaceted intervention, including educational meetings [22]. Supporting our findings, Lagerlov *et al.* tried to distinguish asthma from COPD and found a significant increase of acceptably treated asthma cases after peer-review meetings combined with feedback of prescribing data [10].

Quality circles working with structured feedback have already been shown to improve rational prescribing in general practice [11]. The core element of these circles is the conjoint discussion of evidence-based pharmacotherapy and management of patients on the basis of prescribing data, under the guidance of a trained moderator. Because of the paradigm of ‘multifaceted intervention’[23], these QCs are facilitated with an individual feedback report of prescribing data from each doctor's own practice compared with average data from the QC and from the whole study arm. It remains unclear whether the presentation of ‘best practice of the QC’ and the ‘best 10% of the study arm’ as an achievable benchmark of care can lead to further improvement above the ‘traditional’ means of feedback. One reason for this is that fewer patients participated in the questionnaire survey than initially expected. Therefore, our trial might be too underpowered to detect a specific benchmarking effect. On the other hand, it needs to be taken into account that the traditional feedback report also contains benchmark elements, as one's own practice is compared with certain parameters (median, quartiles, minimum and maximum) from the other GPs in the study arm.

The small increase in self-management behaviours reveals the limitation of QCs. It seems unlikely that more sophisticated performance of QCs would be able to solve this problem, in particular as this kind of patient management is strongly dependent on organization within the practice team. As there is still no ‘magic bullet’[24] in quality improvement, it seems more promising to amplify the multifaceted intervention [23]. Further trials have to show whether accessory outreach visits or academic detailing [25], ideally with the inclusion of the whole practice team, is able to improve these deficits.

Our trial has some limitations. Most important is that only 43 out of 96 practices agreed to distribute the questionnaires because of a high workload due to the implementation of a new fee-for-service structure during the study period. This could have led to a selection of highly motivated GPs and therefore an overestimation of the effectiveness of the QCs. Another critical point is that not all patients responded at follow-up. However, more than 70% did, and the non-responding patients seemed to be slightly healthier. This could also have led to some overestimation of the effectiveness. On the other hand, this underscores the high potential of QCs, as it may have been the more difficult patients who participated.
